# Norwegian consensus guidelines for selection of neuro-oncology patients to proton therapy

**DOI:** 10.1016/j.ctro.2026.101140

**Published:** 2026-02-25

**Authors:** Liv Cathrine Heggebø, Tor-Christian Aase Johannessen, Henriette Magelssen, Mette Sprauten, Hanne Blakstad, Jorunn Brekke, Dorota Goplen, Kirsten Marienhagen, Tora S. Solheim, Line Bjorland, Petter Brandal

**Affiliations:** a Department of Oncology, Oslo University Hospital, Oslo, Norway; b Institute for Clinical Medicine, University of Oslo, Oslo, Norway; c Department of Oncology and Medical Physics, Haukeland University Hospital, Bergen, Norway; d Department of Oncology, University Hospital of North Norway, Tromsø, Norway; e Cancer Clinic, St. Olavs Hospital, Trondheim University Hospital, Trondheim, Norway; f Department of Clinical and Molecular Medicine, Faculty of Medicine and Health Sciences, Norwegian University of Science and Technology, Trondheim, Norway; g Department of Oncology, Stavanger University Hospital, Stavanger, Norway; h Institute for Cancer Genetics and Informatics, Oslo University Hospital, Oslo, Norway

**Keywords:** Radiotherapy, Proton therapy, Guidelines, Patient selection, Brain Neoplasms, Oncology

## Abstract

With the launch of two Norwegian proton facilities, Norwegian Neuro-Oncology Interest Group developed consensus guidelines for selection of neuro-oncology patients to proton therapy. Relevant literature review and nationwide discussions informed the process. An overview of patients offered proton therapy during the first six months of operation was registered.

## Highlights

• Norwegian consensus guideline for neurooncology patient selection to proton therapy.
• Very limited evidence on which patients benefit most from proton therapy.
• Overview of patients offered proton therapy the first months of facility operation.

## Graphical abstract

**Figure:**
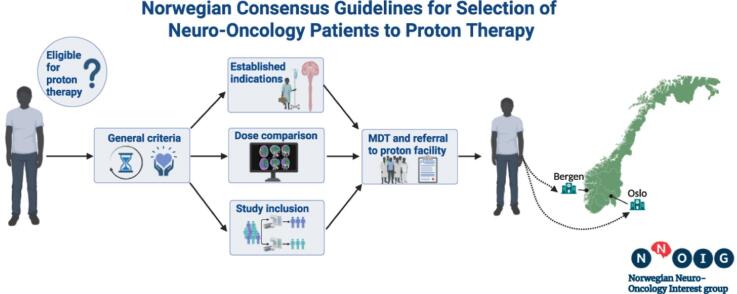

## Introduction

The use of proton beam therapy (PBT) has increased considerably worldwide the last years, yet robust evidence to guide patient selection in neuro-oncology remains limited [1], [2], [3], [4], [5]. Existing data on PBT for intracranial neoplasms demonstrate comparable tumor control rates to photon-based radiotherapy, however, the evidence is primarily derived from small, retrospective, and non-randomized trials with the inherent limitations associated with such designs [3], [6]. Although PBT dose plans often offers superior sparing of organs at risk (OARs) and healthy tissue, it remains uncertain if and to what extent these dosimetric advantages translate into significant clinical benefits [2]. Furthermore, PBT is more costly than conventional photon therapy, is less accessible and thereby often necessitates long-distance travel at a time when patients may already be under substantial physical and emotional strain (“time toxicity”). Careful selection of individuals most likely to benefit from PBT is therefore essential—not only to justify the individual burden of treatment and travel but also from societal and health-economic standpoints [1], [2], [7].

In 2025, two publicly funded PBT facilities fully integrated into the national health‐care system opened in Norway. In these facilities, two rooms are allocated to preclinical research, and three treatment rooms for clinical operations. When fully operational, the estimated annual treatment capacity is approximately 850 patients. Norway spans a large geographical area; consequently patients will have to travel considerable distances to access PBT. Even patients residing in regions with a PBT facility may face long travel times. To ensure equal access to PBT across the country, Norwegian Neuro-Oncology Interest Group (NNOIG) developed national patient selection criteria for PBT in neuro-oncology. Herein, we present the resulting consensus guidelines, and report on the patient population during the first six months of proton facilities’ operation.

## Material and methods

At the initiative of NNOIG, a working and a reference group were established to update and considerably improve national consensus guidelines for patient selection to PBT in neuro-oncology [8]. All involved professionals were experienced clinical neuro-oncologists and NNOIG board members. The working group consisted of three neuro-oncologists; two affiliated with the proton facility at Oslo University Hospital (OUS) and one affiliated with the proton facility at Haukeland University Hospital (HUS). The reference group included eight additional neuro-oncologists from five institutions, representing all health regions in Norway. For the reporting of these guidelines, the AGREE II tool was applied [9] (Supplementary material).

The working group reviewed relevant literature focusing on clinical outcomes, dosimetric studies, and patient selection criteria [4], [6], [10], [11], [12], [13], [14], [15], [16], [17], [18], [19], [20], [21], [22], [23], [24], [25], [26], [27], [28], [29], [30], [31], [32]. Additionally, available information from other proton facilities was reviewed [33], [34], [35], [36], [37], [38], [39], and a draft for subsequent discussions was made. Meetings between the working and reference groups were held before and during the process, beginning in January 2024. The working group corresponded by email and held additional meetings to refine draft recommendations. No formal voting took place; consensus was achieved through expressed agreement or by tacit acceptance. The final guidelines were reviewed and approved by all participants, and finalized and published in February 2025.

We reviewed the number and diagnoses of neuro-oncology patients having received PBT at the two facilities by mid-September 2025, corresponding to six months after opening of the proton facility at OUS and four months following the opening at HUS.

## Results

The Norwegian consensus guidelines for selection of neuro-oncology patients to PBT were formally endorsed by NNOIG prior to the opening of the two national proton facilities. The full guidelines were published in February 2025 on the official NNOIG website and distributed to all involved institutions [40]. It was decided that all patients being considered for PBT should be reviewed in national multidisciplinary team meetings. For all patients, standard OARs according to the European Particle Therapy Network (EPTN) guidelines and the Norwegian national guidelines should be delineated [41], [42], for OARs to be delineated see Table 1.

**Table 1:** Organs at risk to be delineated in proton beam therapy planning.

• Brainstem • SpinalCord • OpticChiasm • OpticNerve_L/R • Eye_L/R • Cornea_L/R • Retina_L/R • Cochlea_L/R • LacrimalGland_L/R • Brain-CTV • Body-CTV • Lens_L/R • Pituitary • Hippocampus_L/R • Hypothalamus_L/R • Periventricular zone
L: Left; R: Right

Given limited strength of current evidence, the guidelines will be revised regularly to incorporate emerging data. Several limitations were acknowledged. First, it was made clear that the guidelines were not intended to replace clinical judgment, but rather to serve as a framework for evidence-informed, consensus-based support for patient selection to PBT. Furthermore, the guidelines focus on adults (≥18 years) considered for fractionated radiotherapy. Moreover, given the presence of >100 primary neuro-oncological neoplastic entities, some of which are extremely rare, it was concluded that developing specific guidelines for each was neither feasible nor practical. Nonetheless, it was unequivocally acknowledged that certain entities not specifically listed also may benefit from PBT.

### Proton selection criteria

All patients considered for PBT must fulfill general eligibility criteria. Furthermore, neuro-oncology entities were stratified into three categories (Table 2):

Established indications
Dose comparison candidates
Patients eligible for study inclusion

**Table 2:** Proton selection criteria.

**General eligibility criteria:** • Age: ≥18 years old • Expected survival: ≥ 5 years • General condition: Eastern Cooperative Oncology Group (ECOG) 0–1/ Karnofsky Performance Status (KPS) 70–100 • Absence of physical, social, or psychological factors hindering the implementation of outpatient radiotherapy. This does not mean that patients with for example neurological deficits cannot be considered for proton therapy if they require hospitalization during treatment • The patient must accept travel to, planning, and treatment at a proton center • Patients with a genetic susceptibility to radiation-related side effects, including but not limited to patients with neurofibromatosis (NF), should be considered for proton therapy if radiation therapy is to be administered
**Established indications:** tumor entities with target volume localization that enables considerable dose sparing of healthy tissue • Patients receiving craniospinal irradiation (CSI) • Spinal tumors with large cranio-caudal target volumes
Dose comparison indications*: entities** (and localizations) requiring individualized photon versus proton dosimetric evaluation to determine optimal treatment modality • Meningiomas CNS WHO grade 1 and 2 (grade 3 can be considered) • Ependymomas (all grades) • Pituitary neuroendocrine tumors • Other sellar tumors (including craniopharyngiomas) • Circumscribed gliomas (including pilocytic astrocytomas) according to the WHO classification • Glioneuronal and neuronal tumors • Pineal tumors • Tumors in cranial and paraspinal nerves (including vestibular schwannomas) • Mesenchymal tumors (including solitary fibrous tumors) • Melanocytic tumors • Germ cell tumors not requiring CSI • Spinal tumors with limited cranio-caudal extension
**Indications eligible for study inclusion:** entities where inclusion in ongoing trials are necessary for proton beam therapy • IDH-mutated gliomas CNS WHO grade 2 and 3 (oligodendrogliomas and astrocytomas) o PRO-GLIO, randomized controlled phase 3 multicenter trial

* Not exhaustive.

** According to the WHO classification of 2021.

### Dose sparing criteria

For patients eligible in the dose comparison category, a set of dose sparing criteria was established (Table 3). Both relative and absolute dose sparing criteria were defined for each evaluated structure, as relying solely on relative sparing may appear favorable despite minimal absolute difference in delivered dose.

**Table 3:** Dose sparing criteria and patient counts per criterion.

Organ/volume	Relative dose reduction (%)*	Absolute dose reduction (Gy (RBE))*	Number of patients selected for proton therapy fulfilling each criterion**
Brain-CTV (D_mean_)	≥ 20	3.0	11
Head-CTV (D_mean_)	≥ 20	3.0	4
Hippocampus_R (D_40%_)	≥ 50	3.0	7
Hippocampus_L (D_40%_)	≥ 50	3.0	9
Cochlea_R (D_mean_)	≥ 50	15	2
Cochlea_L (D_mean_)	≥ 50	15	1
LacrimalGland_R (D_mean_)	≥ 50	10	4
LacrimalGland_L (D_mean_)	≥ 50	10	2
Retina_R (D_0.03cm_^3^)	≥ 50	15	2
Retina_L (D_0.03cm_^3^)	≥ 50	15	1
Cornea_R (D_0.03cm_^3^)	≥ 50	10	0
Cornea_L (D_0.03cm_^3^)	≥ 50	10	1
Pituitary gland (D_mean_)	≥ 50	10	3
CTV: clinical target volume; D_0.03cm_^3^: dose to 0.03 cm^3^; D_40%_: dose received by 40% of volume; D_mean_: mean dose to volume; Gy: Gray; L: left; R: right; RBE: relative biological effect; * For each structure, both the relative and absolute dose reduction criteria must be fulfilled, **most patients fulfilled more than one criterion			

### Brain-CTV, Head-CTV, and periventricular zone

**Fig. 1:**
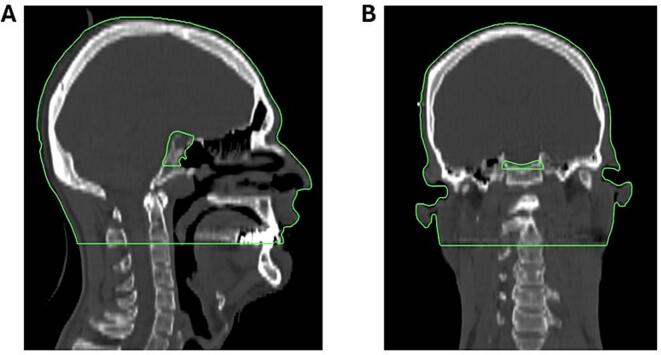
Head minus CTV (Head‐CTV) is defined as the external contour (excluding fixation equipment) cranially from the top of the skull to the caudal dorsal border of dens axis (C2), excluding the CTV itself. Fig. 1 displays an example of Head-CTV (green contour) for a patient with a pituitary neuroendocrine tumor (PitNET), CT dose plan is shown in bone window.

Many neuro-oncological neoplasms may be located or extend extracranially. Brain minus clinical target volume (Brain-CTV) was therefore not viewed as a sufficient parameter alone for evaluating dose to healthy tissue surrounding the target volume. We therefore introduced head minus CTV (Head‐CTV) as a parameter to better account for radiation exposure to healthy tissue beyond the brain parenchyma, such as facial structures. Head-CTV is defined as the external contour cranially from the top of the skull to the caudal dorsal border of dens axis (C2), excluding fixation equipment and the CTV itself (Fig. 1) [28]. Additionally, based on reports of toxicity in the periventricular zone following proton therapy, it was decided that this structure should be delineated in all patients considered for PBT.

### Ongoing trials

Before the opening of the two Norwegian proton facilities, there was a strong ambition to include a substantial proportion of patients treated with proton therapy in clinical trials. For neuro-oncological patients, the only ongoing trial at the initiation of PBT in Norway was the PRO-GLIO trial. PRO-GLIO is a randomized controlled multicentre phase 3 trial for patients with isocitrate dehydrogenase (*IDH*)-mutated gliomas CNS WHO grade 2 and 3 (oligodendrogliomas and astrocytomas). Eligible patients are randomized 1:1 to receive radiotherapy delivered with photons (standard arm) or protons (experimental arm). A total of 224 patients from Norway and Sweden will be enrolled and followed for 15 years. The primary endpoint is first intervention free survival (FIFS) at two years, and several secondary and exploratory endpoints will also be assessed [31]. Several additional initiatives are underway to develop PBT trials for other neuro-oncological neoplastic entities.

### Individual plan evaluations using the guidelines

By mid-September 2025 a total of 28 adult neuro-oncological patients had been treated with PBT in Norway. Of these, nine had received PBT through randomization in the PRO-GLIO trial [31], two patients received PBT based on established indications, and 21 patients had been discussed as dose comparison candidates. Of the latter, 17 (71%) were selected to PBT based on the dosimetric criteria. The most frequently met dose-sparing criterion was dose reduction to brain-CTV, achieved in 11 patients. Among the selected patients, five fulfilled one criterion, two fulfilled two criteria, and nine fulfilled three or more criteria, in addition, one patient did not fulfill any criterion, but nearly fulfilled several criteria and was considered to likely benefit from PBT.

Discussion and conclusion

Although PBT is considered safe and theoretically beneficial in comparison to conventional photon therapy, especially considering long-term toxicity [3], [43], it remains uncertain if and how much the proton-enabled dose sparing of healthy tissue translates into clinical benefits [3], [6]. Consequently, robust data guiding patient selection for PBT is still limited. These national guidelines are limited to adult patients and do not specify all neuro-oncological entities. Importantly, the guidelines divide neuro-oncological entities/localizations into three PBT selection categories and introduce the new parameter Head-CTV.

PBT will in most cases be dosimetrically superior to photon therapy. Nonetheless, photon therapy techniques have improved substantially over the last decades, thereby reducing the dosimetric superiority of protons. Also, uncertainties related to PBT, such as relative biological effect (RBE) and linear energy transfer (LET), still warrant further research [44]. For example, LET effects may lead to higher doses than anticipated in some areas, thereby potentially increasing the risk of toxicity. This pertains especially to critical serial OARs such as the optic nerves, chiasm, and brainstem. Several reports on unexpected toxicity following PBT might have been influenced by such factors. The latter include radiation-induced contrast enhancement (RICE), although it is unclear if RICE incidence differs between protons and photons [45], [46], [47], [48]. We strongly encourage interventional studies and head-to-head comparison on proton versus photon therapy, and emphasize that PBT patient selection guidelines must take these uncertainties into account. International collaboration on toxicity evaluation is ongoing, and several initiatives exploring LET-weighted dose are currently in progress. Members from the Norwegian proton community are also partaking in several EPTN working groups. In addition to other measures, we believe that a structured and uniform follow-up is critical for identifying treatment-related toxicity following PBT. A national Norwegian effort is underway to further standardize these procedures.

The presumed reduction of long-term side effects is considered the most paramount advantage of PBT. Accordingly, our guidelines emphasized general criteria such as good overall health and long life expectancy as prerequisites for offering PBT, well in line with other PBT selection guidelines [33], [34], [35], [36], [37], [38]. As some studies suggest that PBT is beneficial also for elderly patients [49], we have not included an upper age limit in our guidelines. However, a lifetime expectancy of >5 years is required. We also chose to include genetic vulnerability among the general criteria, as any dose reduction is more likely to be beneficial for these patient groups [50].

In our category of established indications for PBT, CSI is the most prominent. This is well aligned with existing recommendations due to the large irradiated volume and the substantial dose reduction to numerous OARs [12], [14], [27], [32]. Following the same rationale, spinal tumors with large cranio-caudal target volumes were defined as an established PBT indication.

The largest guideline category is patients considered for PBT based on dose comparison of proton versus photon plans. To allow for this, we needed evaluation criteria. Instead of criteria based on normal tissue complication probability, we defined criteria based on dose to OARs. Which OARs to include for evaluation was discussed when settling for the dose sparing criteria, and it was decided that the brainstem, chiasm, and optic nerves should not be included. Fulfilling one criterion is sufficient to be offered PBT, however, the evidence supporting each criterion is scarce, and they should therefore be used with caution. An unwanted effect of the criteria might be redistribution of dose to areas not defined as OARs, such as the frontal lobes, which is undesirable. Also, we chose to include an absolute dose reduction alongside relative dose sparing, in contrast to some PBT facilities that have chosen to assess only relative dose reduction, which may select patients to PBT based on a very low dose reduction in absolute terms. Furthermore, we introduce Head-CTV to enable assessment of dose sparing to all healthy tissue, not only brain tissue. For Brain-CTV and Head-CTV we chose a relative dose reduction of ≥20% in addition to an absolute dose reduction of 3.0Gy (RBE). These thresholds are debatable as doses as low as 1-2 Gy have been implied to increase the risk of developing secondary neoplasms [51]. It is, however, hard to conclude whether secondary neoplasms develop in low or high dose areas, the effects of LET from protons in this matter are unknown, and secondary neoplasms are very rare and usually occur decades after radiotherapy [52]. A recent paper suggests that a mean brain dose >3 Gy is associated with cognitive decline, potentially supporting our absolute criterium to Brain-CTV. However, given low-quality evidence, further research and individualized evaluation of each patient’s situation remain necessary for the selection of proton therapy [53].

The third category in these guidelines are interventional studies comparing proton and photon therapy. As of now, this applies only to oligodendrogliomas and astrocytomas CNS WHO grade 2 and 3 [31]. Based on available, but limited published data, we believe that meningioma patients, in addition to other neoplastic entities, will benefit from inclusion in clinical studies.

Our experience to date indicates that the criteria are generally adhered to. Nonetheless, borderline cases do arise. For example, in patients that fulfil some criteria favoring PBT, other criteria might favor photons. In other cases, a patient may not fulfill any selection criteria, but nearly fulfill multiple criteria, and thereby be considered to likely benefit from PBT. In addition, all OAR dose reductions should be weighted individually as the potential clinical relevance is likely higher reducing the dose from 10 Gy to 5 Gy, compared to 55 Gy to 50 Gy. We believe, radiotherapy is not a binary choice between protons or photons; individualized planning is imperative to choose the optimal treatment.

In Norway, radiotherapy is fully publicly funded and equal access to PBT is considered important, differing from some countries where access may be influenced by the patients’ health insurance [36], [38]. Increasing knowledge on effects and side effects from PBT is imperative to be able to advise patients optimally. More knowledge will enable patients to choose based on subjective preferences and avoid unnecessary time toxicity. Hopefully, these national consensus guidelines may contribute to better patient selection and pave the way for relevant interventional studies.

In summary, reflecting the current lack of high-quality data on neuro-oncology patient selection to PBT, we do not consider these guidelines strictly evidence-based, however, intend them to represent a pragmatic support for clinical decision-making. The guidelines also aim to provide equal access to PBT for all eligible neuro-oncology patients. Further interventional studies are needed. Generating robust evidence, along with full transparency on possible benefits and uncertainties related to PBT, is key to optimal patient counselling and justification of resource allocation.

## Ethics declarations & trial registry information

Not applicable.

## Funding

This research did not receive any specific grant from funding agencies in the public, commercial, or not-for-profit sectors.

## Declaration of competing interest

The authors declare that they have no known competing financial interests or personal relationships that could have appeared to influence the work reported in this paper.
